# Facial Attractiveness and Group Identity Influence Decision‐Making

**DOI:** 10.1002/pchj.70004

**Published:** 2025-02-24

**Authors:** Junchen Shang, Kaiyin Zhong

**Affiliations:** ^1^ School of Humanities Southeast University Nanjing Jiangsu China

**Keywords:** beauty premium, facial attractiveness, fairness, group identity, ultimatum game

## Abstract

This study examined the impact of facial attractiveness and group identity of male proposers on the fairness decision‐making of female participants in an ultimatum game. Results showed that participants were more likely to accept unfair offers from both attractive proposers and in‐group proposers.

## 1 Introduction

Human decisions are impacted by many factors like facial attractiveness and group identity. Attractive faces increased the acceptance rate of unfair offers in the ultimatum game (Pan et al. 2022). However, the influence of group identity on decision‐making remains inconsistent. Social identity theory (SIT) suggests people are more tolerant of unfair offers from in‐group members, that is, in‐group favoritism (IGF; Valenzuela and Srivastava 2012). Nonetheless, norm‐focused theory (NFT) suggests people reject unfair offers from in‐group members more frequently because of the aversion to violating in‐group rules (Mendoza et al. 2014), that is, the black sheep effect (BSE). Previous research explored the impact of facial attractiveness or group identity on decision‐making separately. However, attractiveness ratings may change with group identity. Zhao et al. (2012) found that participants rated out‐group members' faces as more attractive compared with in‐group members, even though attractiveness ratings of the two groups of faces did not differ before the experiment. Therefore, it is unclear whether the influence of attractiveness on fairness decisions would be modulated by group identity.

Gender also modulated facial attractiveness effect in decision‐making. Nevertheless, this experiment was too complex to include gender variables (face gender and participant gender). We recruited female participants and used male faces (Shang and Zhang 2024) to investigate the joint effects of facial attractiveness and group identity on ultimatum game decision‐making.

## 2 Method

Fifty‐five female students (*M*_age_ = 23.15 years, SD = 1.88) from Southeast University participated in the experiment approved by the Ethics Committee of the Psychology Research Center at Southeast University. Each participant provided written informed consent.

This was a 2 (facial attractiveness: attractive, unattractive) × 5 (fairness: 9:1, 8:2, 7:3, 6:4, 5:5) × 2 (group identity: in‐group, out‐group) within‐participant design. Sixty attractive and sixty unattractive male faces with a neutral expression (Shang and Zhang 2024) were used. Independent samples *t*‐tests were conducted on trustworthiness, pleasure, arousal, and dominance ratings of the two sets of faces (Data S1. Detailed statistics are also available at https://osf.io/yxk6h/). Only the difference in attractiveness ratings was significant, *p* < 0.001. Among each set, half of the faces were assigned to the in‐group condition.

First, using the minimal group paradigm (MGP), participants were classified into red/blue personality groups via a sham personality test (Wu et al. 2015). Then, they were told the proposer would allocate ¥10 between himself and the participant, who could accept/reject the offer. If the offer was accepted, they would receive the proposed amount of money; otherwise, they would receive nothing. To facilitate manipulation of group identity, participants were told the higher‐income group would win a bonus (Rilling et al. 2008). Each trial began with a central fixation (400–600 ms). Then, a proposer's face was presented on a red/blue background (2000 ms), followed by an interval (200–300 ms). A monetary allocation was then presented, and the participant decided to accept/reject it. There was an interval after the response (200–300 ms). Then, the final outcome was presented (2000 ms). Each face was paired with one fairness level, with 120 trials presented in a random order. Twenty practice trials preceded the experiment. After the experiment, participants rated the facial attractiveness on a 7‐point scale (1 = unattractive, 7 = attractive).

## 3 Results

**FIGURE 1:**
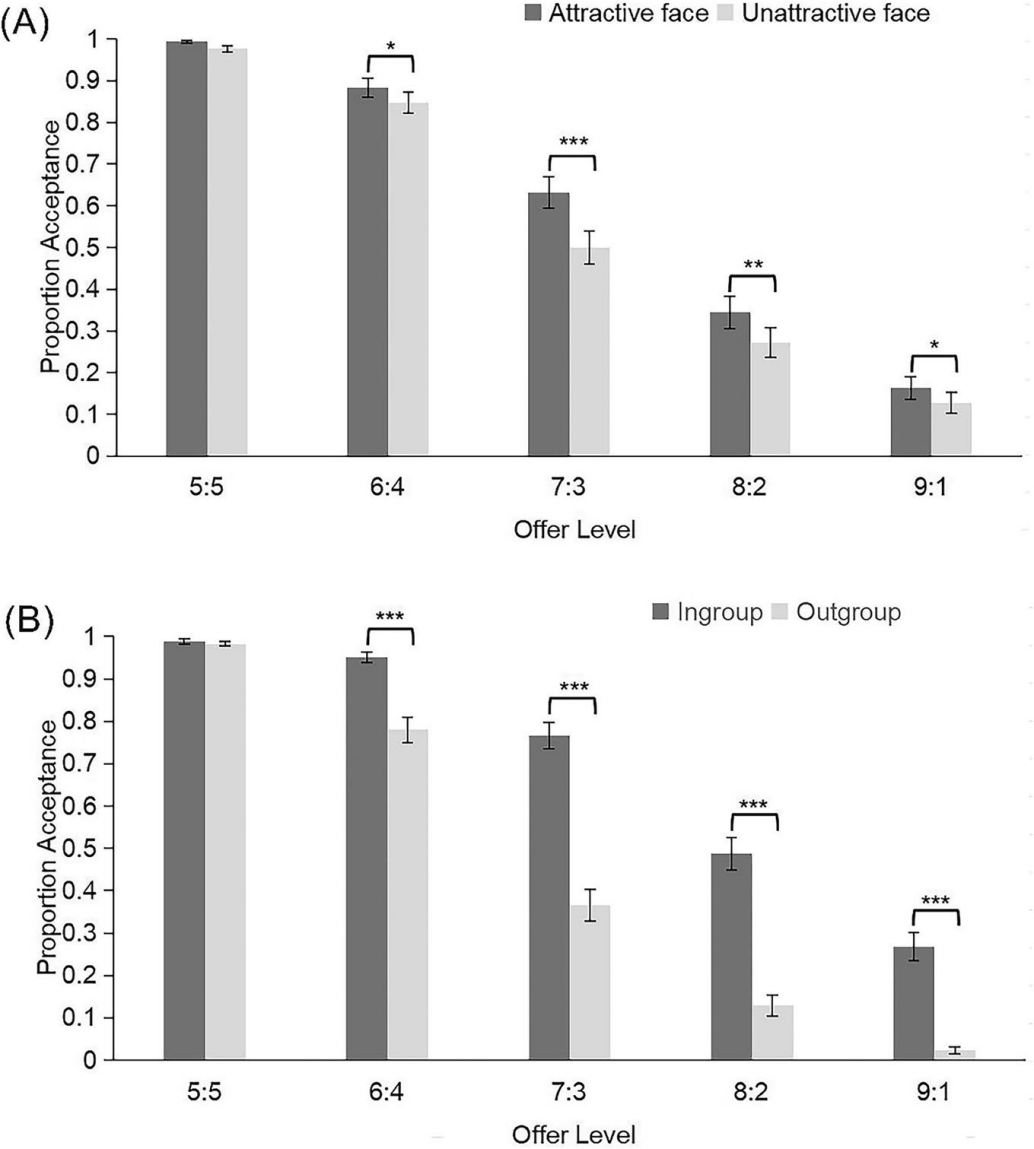
(A) Mean proportion acceptance as a function of offer fairness and facial attractiveness. (B) Mean proportion acceptance as a function of offer fairness and group identity. Error bars represent standard errors. *p < 0.05, **p < 0.01, ***p < 0.001.

Three‐way repeated measures ANOVA was performed on the acceptance rate. Greenhouse–Geisser correction was applied for sphericity departures. All post hoc analyses were Bonferroni‐corrected. The main effects of facial attractiveness, fairness, and group identity were significant, *F*s ≥ 27.383, *p*s < 0.001. The interaction between facial attractiveness and fairness was significant (Figure 1A), *F*(3, 157) = 7.707, *p* < 0.001, *η*_p_^2^ = 0.125. Simple effect analysis showed that the acceptance rate of unfair offers from attractive proposers was significantly higher than that from unattractive proposers, *F*s ≥ 4.929, *p*s ≤ 0.031, except for offer 5:5, *F*(1, 54) = 3.414, *p* = 0.07. The interaction between group identity and fairness was significant (Figure 1B), *F*(3, 170) = 21.459, *p* < 0.001, *η*_p_^2^ = 0.284. Simple effect analysis showed that the acceptance rate of unfair offers from in‐group proposers was significantly higher than that from out‐group proposers, *F*s ≥ 19.145, *p*s < 0.001, except for offer 5:5, *F*(1, 54) = 0.424, *p* = 0.518. Other effects were not significant, *F*s ≤ 1.453, *p*s ≥ 0.23.

Post‐experimental attractiveness ratings showed that attractive faces (*M* = 4.30, SD = 0.67) were rated as more attractive than unattractive faces (*M* = 1.74, SD = 0.28), *t* (79) = 27.049, *p* < 0.001, 95% CI = [2.36, 2.74].

## 4 Discussion

Results indicated that participants accepted more unfair offers from attractive proposers rather than those from unattractive proposers, consistent with previous studies (Pan et al. 2022), revealing a beauty premium effect in which the reward value of attractive faces attenuated inequality feelings. Moreover, participants were more likely to accept unfair offers from in‐group proposers, consistent with previous research showing that participants accepted more unfair offers from in‐group proposers and were angrier toward unfair offers from out‐group proposers (Valenzuela and Srivastava 2012), supporting SIT. However, we observed no interaction between facial attractiveness and group identity in the acceptance rate of unfair offers. One potential explanation is that negative feelings caused by unfair offers may lead to narrowed attention (Derryberry and Reed 1998). It is challenging for participants to consider both factors simultaneously.

It should be noted that cross‐sex design (female participants and male faces) limited the generalizability of our findings to other genders' decisions. Future research should examine the role of facial attractiveness and group identity in more gender samples. Additionally, MGP lacks ecological validity versus real‐world group identity. This possibly explains why we did not observe BSE since some researchers reported BSE using race (black/white) to create groups (Mendoza et al. 2014). Future research should use real social identity to explore whether BSE exists.

## Ethics Statement

The research was approved by the Ethics Committee of the Psychology Research Center at Southeast University. Each participant provided written informed consent.

## Conflicts of Interest

The authors declare no conflicts of interest.

## Supporting information


**Data S1.** Supporting Information.
