# Supplementary material for: Facial Attractiveness and Group Identity Influence Decision‐Making
Source: Psych J. 2025 Feb 24;14(3):457–9. doi: 10.1002/pchj.70004 (PMC12133228; doi:10.1002/pchj.70004)
Supplement: Supplementary file 1 — Data S1. Supporting Information. [file PCHJ-14-457-s001.docx]

*“The facial stimuli were selected from the male face database of our previous study, which does not contain movie stars, musicians, or other celebrities (Shang & Zhang, 2024). All face images were with neural expression and were processed to a uniform size and grayscale by Adobe Photoshop 2022, such that the interference of additional features (e.g., neck, clothes, etc.) was removed. 52 university students (26 males, M_age_ = 21.25 years, SD = 2.13) were recruited to rate the faces on a 9-point scale for 5 dimensions (attractiveness, trustworthiness, pleasure, arousal, and dominance). After sorting the face materials according to attractiveness scores, and comprehensively considering the number of face materials and the need of this study, we selected 60 attractive and 60 unattractive male faces as the formal experimental materials (see Table 1 for the descriptive statistics of the ratings). Independent samples T-tests were conducted on the ratings of each dimension for these two groups of faces. Attractive faces were rated as more attractive compared to unattractive faces, t(91) = 34.95, p < 0.001, 95% CI [2.58, 2.89]. But the ratings of trustworthiness (t(118) = 1.51, p = 0.125, 95% CI [-0.06, 0.41]), pleasure (t(118) = 1.73, p = 0.201, 95% CI [-0.03, 0.40]), arousal (t(118) = 1.73, p = 0.079, 95% CI [-0.02, 0.27]) and dominance (t(118) = 0.35, p = 0.684, 95% CI [-0.06, 0.09]) were not significantly different between the two groups of faces.” We hope these revisions meet your expectations.*

*Table 1. Means and standard deviations of ratings of facial attractiveness, trustworthiness, pleasure, arousal and dominance (M ± SD).*

|  | **Attractiveness** | | **Trustworthiness** | | **Pleasure** | | **Arousal** | | **Dominance** | |
| --- | --- | --- | --- | --- | --- | --- | --- | --- | --- | --- |
|  | ***M*** | ***SD*** | ***M*** | ***SD*** | ***M*** | ***SD*** | ***M*** | ***SD*** | ***M*** | ***SD*** |
| Attractive faces | 5.67 | 0.53 | 4.80 | 0.70 | 4.52 | 0.63 | 4.14 | 0.45 | 5.73 | 0.23 |
| Unattractive faces | 2.93 | 0.29 | 4.62 | 0.59 | 4.33 | 0.54 | 4.01 | 0.35 | 5.72 | 0.20 |

*Reference:*

*Shang, J., & Zhang, Y. (2024). Influence of male’s facial attractiveness, vocal attractiveness and social interest on female’s decisions of fairness. Scientific Reports, 14(1), 16778.*
